# Polyphenol-rich *Salicornia* extract in lacunar stroke: a pilot randomised trial of safety and exploratory clinical outcomes

**DOI:** 10.1093/esj/aakag049

**Published:** 2026-05-20

**Authors:** Soledad Pérez-Sánchez, Carmen del Río, David Núñez-Jurado, Ana María Najar, Rafael F Ocete, Cristina López Azcárate, Carmen Domínguez-Ruiz, Reyes de Torres Chacon, Ana Barragán-Prieto, Ana Domínguez-Mayoral, Alexandra Sevilla-Bravo, José Luis Fernández-García, Eleonora Magni, Teresa Busquier, Antonio León-Justel, Joan Montaner

**Affiliations:** Department of Neurology, Hospital Universitario Virgen Macarena, Sevilla, Spain; Neurovascular Research Group, Instituto de Biomedicina de Sevilla, IBiS/Hospital Universitario Virgen Macarena/CSIC/Universidad de Sevilla, Sevilla, Spain; Neurovascular Research Group, Instituto de Biomedicina de Sevilla, IBiS/Hospital Universitario Virgen Macarena/CSIC/Universidad de Sevilla, Sevilla, Spain; Department of Biochemistry, Hospital Universitario Virgen Macarena, Sevilla, Spain; Department of Neurology, Hospital Universitario Virgen Macarena, Sevilla, Spain; Neurovascular Research Group, Instituto de Biomedicina de Sevilla, IBiS/Hospital Universitario Virgen Macarena/CSIC/Universidad de Sevilla, Sevilla, Spain; Department of Biochemistry, Hospital Universitario Virgen Macarena, Sevilla, Spain; Department of Neurology, Hospital Universitario Virgen Macarena, Sevilla, Spain; Neurovascular Research Group, Instituto de Biomedicina de Sevilla, IBiS/Hospital Universitario Virgen Macarena/CSIC/Universidad de Sevilla, Sevilla, Spain; Neurovascular Research Group, Instituto de Biomedicina de Sevilla, IBiS/Hospital Universitario Virgen Macarena/CSIC/Universidad de Sevilla, Sevilla, Spain; Department of Radiology, Hospital Universitario Virgen del Rocío, Sevilla, Spain; Department of Neurology, Hospital Universitario Virgen Macarena, Sevilla, Spain; Neurovascular Research Group, Instituto de Biomedicina de Sevilla, IBiS/Hospital Universitario Virgen Macarena/CSIC/Universidad de Sevilla, Sevilla, Spain; Department of Neurology, Hospital Universitario Virgen Macarena, Sevilla, Spain; Neurovascular Research Group, Instituto de Biomedicina de Sevilla, IBiS/Hospital Universitario Virgen Macarena/CSIC/Universidad de Sevilla, Sevilla, Spain; Department of Neurology, Hospital Universitario Virgen Macarena, Sevilla, Spain; Neurovascular Research Group, Instituto de Biomedicina de Sevilla, IBiS/Hospital Universitario Virgen Macarena/CSIC/Universidad de Sevilla, Sevilla, Spain; Department of Neurology, Hospital Universitario Virgen Macarena, Sevilla, Spain; Neurovascular Research Group, Instituto de Biomedicina de Sevilla, IBiS/Hospital Universitario Virgen Macarena/CSIC/Universidad de Sevilla, Sevilla, Spain; Department of Neurology, Hospital Universitario Virgen Macarena, Sevilla, Spain; Neurovascular Research Group, Instituto de Biomedicina de Sevilla, IBiS/Hospital Universitario Virgen Macarena/CSIC/Universidad de Sevilla, Sevilla, Spain; Department of Neurology, Hospital Universitario Virgen Macarena, Sevilla, Spain; Neurovascular Research Group, Instituto de Biomedicina de Sevilla, IBiS/Hospital Universitario Virgen Macarena/CSIC/Universidad de Sevilla, Sevilla, Spain; Neurovascular Research Group, Instituto de Biomedicina de Sevilla, IBiS/Hospital Universitario Virgen Macarena/CSIC/Universidad de Sevilla, Sevilla, Spain; Faculty of Nursing, Physiotherapy and Podology, University of Seville, Sevilla, Spain; Department of Radiology, Hospital Universitario Virgen Macarena, Sevilla, Spain; Department of Biochemistry, Hospital Universitario Virgen Macarena, Sevilla, Spain; Department of Neurology, Hospital Universitario Virgen Macarena, Sevilla, Spain; Neurovascular Research Group, Instituto de Biomedicina de Sevilla, IBiS/Hospital Universitario Virgen Macarena/CSIC/Universidad de Sevilla, Sevilla, Spain

**Keywords:** cerebral small vessel disease, lacunar stroke, polyphenols, vascular cognitive impairment

## Abstract

**Introduction:**

Cerebral small vessel disease is a major cause of lacunar stroke and vascular cognitive impairment, for which effective disease-modifying treatments are lacking. Polyphenols have shown neurovascular and neuroprotective effects in experimental models. We conducted a randomised pilot trial primarily to evaluate the safety and tolerability of a polyphenol-rich *Salicornia* extract in patients with lacunar stroke.

**Patients and methods:**

In this randomised, double-blind, placebo-controlled, parallel-group pilot trial, patients with recent lacunar stroke received either 1 g/day of a polyphenol-rich *Salicornia* extract or placebo for 12 months (ClinicalTrials.gov NCT06076122). The primary endpoint was safety and tolerability. Secondary exploratory outcomes included cognitive performance (Montreal Cognitive Assessment), physical function (6-min walk test and gait speed), biochemical parameters, ambulatory blood pressure and neuroimaging markers, assessed at baseline, 6 months and study completion.

**Results:**

Seventy-one participants were randomised (40 to placebo and 31 to *Salicornia* extract). No treatment-related serious adverse events were observed. Exploratory analyses of secondary outcomes suggested possible between-group differences in some cognitive and physical measures. At 12 months, adjusted MoCA scores were higher in the *Salicornia* group than in the placebo group (adjusted mean difference 2.22 points; 95% CI, 0.08–4.36; *P* = .042), although cognitive data were available for only 22/40 placebo-treated and 19/31 *Salicornia*-treated participants. Apparent differences were also observed in some physical function measures during follow-up. In biochemical parameters, a reduction in homocysteine levels was observed in both groups. No relevant between-group differences were detected in neuroimaging markers during follow-up. These secondary findings should be interpreted as exploratory only and not as evidence of efficacy.

**Discussion:**

Any apparent differences in secondary exploratory cognitive, functional and biochemical outcomes should be interpreted cautiously because of baseline imbalances, small sample size and substantial missing follow-up data. These findings should be considered hypothesis-generating only and not as evidence of efficacy.

**Conclusions:**

Long-term supplementation with a polyphenol-rich S ramosissima extract showed an acceptable safety profile in patients with lacunar stroke, although tolerability should be interpreted cautiously given the withdrawal rate. Larger, adequately powered trials are needed to determine whether these preliminary findings represent true treatment effects.

## Graphical Abstract

**Graphical Abstract:**
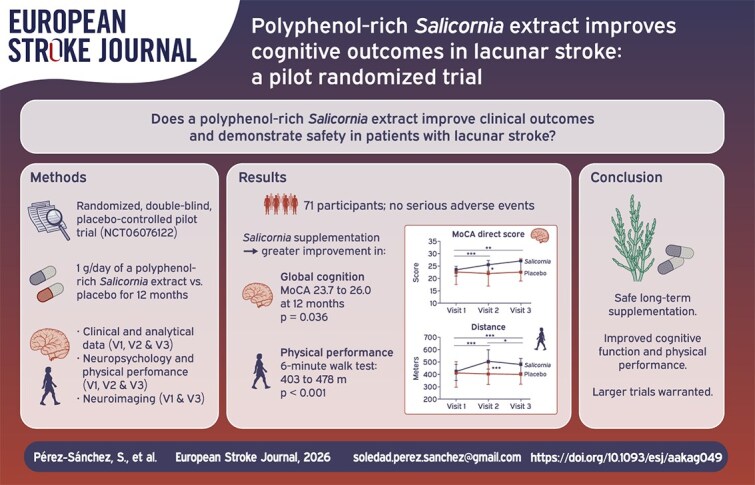

## Introduction

Cerebral small vessel disease (SVD) is a major contributor to vascular cognitive impairment and dementia and is closely associated with lacunar and haemorrhagic stroke. Lacunar infarctions account for approximately one-quarter of all ischaemic strokes and typically present as small (<15 mm) subcortical lesions resulting from pathology of a single perforating artery and intrinsic microvascular abnormalities.^1^ Beyond its role in lacunar stroke and intracerebral haemorrhage, SVD represents the most prevalent underlying substrate of vascular cognitive impairment and constitutes a major driver of dementia in older adults in whom mixed neurodegenerative and vascular aetiologies frequently coexist.^2^ Recent European Stroke Organisation (ESO) guidelines highlight the persistent lack of effective evidence-based strategies for secondary prevention in lacunar stroke, with available data largely derived from small subgroups and rated as moderate to low quality.^3^ In this context, the Lacunar Intervention Trial-2 demonstrated the potential of alternative pharmacological approaches, such as isosorbide mononitrate and cilostazol, to improve functional dependency, cognitive trajectories and quality of life.^4^

Among modifiable determinants of SVD, dietary habits have emerged as key factors influencing long-term cerebrovascular health. The Mediterranean diet provides the most consistent and robust evidence for the prevention of vascular disease and age-related cognitive decline.^5^ Within this dietary framework, polyphenols, widely distributed bioactive compounds, have attracted increasing attention for their neuroprotective properties. Accumulated evidence suggests that polyphenols mitigate ischaemic injury by reducing oxidative stress, modulating inflammatory pathways, preserving endothelial function and regulating cell-death mechanisms, thereby contributing to neurovascular protection.^6,7^

*Salicornia*, an annual halophyte widely distributed along European coastlines, is a rich source of bioactive phytochemicals. More than 60 distinct polyphenolic compounds have been identified in *Salicornia*, many of which are synthesised in response to environmental stressors such as high soil salinity. These polyphenols have attracted interest for their antioxidant and anti-inflammatory properties. In preclinical studies, polyphenol-rich *Salicornia* extracts demonstrated neuroprotective effects and significantly reduced tissue damage in experimental models of cerebral ischaemia.^7,8^ Moreover, we recently showed that the extract was safe and well tolerated in healthy volunteers^9^ and observed favourable trends in vascular risk factors when administered to patients with transient ischaemic attack or minor stroke.^10^ In these previous clinical trials, we also observed a reduction in homocysteine levels, a biomarker strongly associated with cognitive impairment and SVD progression, further supporting its potential relevance in this patient population.^11^

Building on this background, the present study investigates the safety, tolerability and potential clinical benefits of a polyphenol-rich extract in patients with lacunar stroke. We hypothesised that this nutraceutical intervention may improve vascular risk profiles, cognitive performance, gait and biochemical biomarkers, offering a complementary therapeutic strategy for small vessel cerebrovascular disease. Given the lack of disease-modifying therapies for cerebral SVD, exploratory pilot trials may provide important early signals regarding novel therapeutic strategies.

## Patients and methods

### Study design and ethical considerations

A randomised, double-blind, parallel-group, placebo-controlled pilot trial was conducted to evaluate the safety and tolerability of a food supplement based on a *Salicornia* plant extract in patients with lacunar stroke. The primary endpoint of the study was safety and tolerability of long-term supplementation with *Salicornia* extract.

Secondary exploratory endpoints included biochemical markers, ambulatory blood pressure parameters, cognitive performance, physical function and neuroimaging variables. The study was performed in accordance with the Code of Ethics of the World Medical Association (Declaration of Helsinki) for research involving human subjects and was registered at *clinicaltrials.gov* (identifier: NCT06076122). Ethical approval was obtained from the *Andalusian Ethics Committee* (ID HALOFITAS). The trial was conducted at Virgen Macarena University Hospital (Seville, Spain). Written informed consent was obtained from all patients or their legal representatives. This manuscript adheres to the applicable Consolidated Standards for Reporting Trials (CONSORT) guidelines. The present manuscript reports the results of the lacunar stroke sub-study within the broader registered project evaluating polyphenol-rich *Salicornia ramosissima* extract in different populations, including healthy volunteers and patients with transient ischaemic attack or minor stroke, whose results have been reported elsewhere.^9,10^

### Intervention and placebo

Fresh *S ramosissima* plants, cultivated in a salt marsh on the coast of Huelva, Spain, were harvested, dried and processed to obtain hydroalcoholic extracts by EVESA (Cadiz, Spain [https://evesa.com/]). The manufacturing process included preparation of the plant material, extraction with hydroalcoholic solvents and subsequent separation of the liquid and solid phases. The obtained extract is then concentrated by vacuum drying under controlled thermal conditions to maintain product stability, optimise yield and preserve heat-sensitive bioactive constituents. Adequate masking of the intervention was ensured by using a placebo identical in appearance to the active supplement. Encapsulation and blinding procedures were performed by BIO-DIS laboratories (Seville, Spain [https://www.bio-dis.com/]), certified specialists in food supplements.

Each capsule (1 g) contained 0.5 g of *Salicornia* extract combined with a filling agent. Placebo capsules contained only the filling agent, without the extract. Participants received 2 capsules daily for 12 months. The total polyphenol content of each *Salicornia* extract capsule was 12 ± 2 mg of gallic acid equivalent/g (Nutritional composition of *S ramosissima* hydroalcoholic extract was described in Romero-Bernal et al. Food and Chemical Toxicology, 2025).^12^ Placebo capsules were identical in appearance, taste and colour to the active *Salicornia* extract capsules to ensure adequate blinding of participants, investigators and outcome assessors. Each treatment pack was assigned a unique participant code, preventing differentiation between supplement and placebo. This code became the subject’s study identifier.

Randomisation was performed in a 1:1 ratio using a computer-generated allocation sequence prepared by an independent investigator not involved in patient recruitment or outcome assessment. Given the exploratory pilot nature of the study, simple randomisation without blocking or stratification was applied. In relatively small samples, this approach may lead to unequal group sizes by chance. Allocation concealment was ensured through centralised management by the hospital pharmacy, which prepared sequentially coded treatment packages. Investigators, participants and outcome assessors remained blinded throughout the study.

Patients were randomly assigned in a 1:1 ratio to receive either a polyphenol-rich extract or placebo, with clinical assessments at baseline (visit 1), 6 months (visit 2) and at the study’s conclusion (visit 3) at 12 months. All participants received standard medical care for secondary stroke prevention according to current international guidelines, including those of the European Stroke Organisation and the American Heart Association/American Stroke Association.

Adherence to the intervention was assessed by capsule return counts at study visits. As 360 capsules were dispensed for each 6-month period, adherence was estimated from the number of capsules returned at follow-up visits.

### Eligibility criteria

Eligible participants were adults (≥18 years) diagnosed with lacunar syndrome and presenting an acute focal ischaemic lesion of less than or equal to 20 mm in maximum diameter on neuroimaging, who were willing and able to provide written informed consent. Patients were eligible if they had experienced a clinically evident lacunar stroke within the previous 24 months. This time window was chosen to allow inclusion of patients in a stable post-stroke phase while minimising the influence of acute recovery processes. Exclusion criteria included the use of nutritional supplements containing vitamins or polyphenols within 30 days prior to screening; hyperthyroidism as determined by the investigator; claustrophobia or morbid obesity (body mass index [BMI] > 40); precluding 3 T magnetic resonance imaging (MRI); dependency in basic activities of daily living (mRS score > 3) or the presence of severe comorbidities with an expected life expectancy < 12 months; dysphagia interfering with study treatment intake; known allergy or intolerance to halophyte plants; pregnancy or lactation; active neoplastic disease; participation in another clinical trial with investigational drugs within 30 days prior to screening or plans to do so during the study period; habitual consumption of halophyte plants or any other condition that, in the opinion of the investigator, could interfere with protocol adherence or study participation.

Study withdrawal criteria were also established: presence of allergy or intolerance related to the treatment, revocation of informed consent, loss of follow-up, non-compliance by the patient with the study protocol, occurrence of pregnancy or death.

### Safety evaluation

Adverse events (AEs) were systematically monitored during in-person visits and interim telephone contacts using a dedicated study line. All AEs were documented in medical records and Case Report Forms, coded with MedDRA (v25.0) and classified by system organ class, causality and severity. Serious AEs included death, life-threatening events, hospitalisation, prolonged stay or significant disability. Only AEs considered potentially related to the investigational product were analysed in detail, and their frequency and impact on study withdrawal were determined.

### Efficacy evaluation

All study assessments were performed by personnel blinded to treatment allocation. Clinical evaluation, laboratory analyses, ambulatory blood pressure monitoring, cognitive testing and physical function assessments were conducted at all 3 study visits (baseline, 6 months and 12 months). Brain MRI was performed at baseline and repeated at the final visit.

#### Blood sampling and analytical evaluation

At baseline (visit 1) and during visits 2 and 3, analytical studies were performed. After an overnight fast, venous blood samples were drawn between 8:00 and 11:00 AM. Two tubes were collected: one containing an ethylenediaminetetraacetic acid (EDTA) anticoagulant (BD Vacutainer K2EDTA spray-coated) for plasma cell counts using a Sysmex XN-2000 analyser (Sysmex, Kobe, Japan), and another without anticoagulant (BD Vacutainer PST) for serum biochemical analyses using the Alinity analyser (Abbott Laboratories, Abbott Park, IL).

General biochemical parameters were assessed, including lipid profile (total cholesterol, high-density lipoprotein [HDL], low-density lipoprotein [LDL] and triglycerides); liver enzymes (aspartate aminotransferase, alanine aminotransferase and gamma-glutamyltransferase); specific proteins and metabolites (C-reactive protein [CRP], homocysteine, vitamin B12, folate, glucose, albumin and glycated haemoglobin [HbA1c]); electrolytes (sodium and potassium); renal function parameters (serum creatinine and estimated glomerular filtration rate, calculated using the Chronic Kidney Disease Epidemiology Collaboration equation) and thyroid-stimulating hormone (TSH).

#### Ambulatory blood pressure monitoring

Ambulatory blood pressure monitoring was performed using the validated OnTrak device (Spacelabs). Measurements were obtained outside the hospital setting over a 24-h period following the study visit. Data recorded included systolic blood pressure (SBP, mmHg), diastolic blood pressure (DBP, mmHg), mean pulse pressure (mmHg), mean arterial pressure (MAP, mmHg), heart rate (beats per minute, bpm), morning surge index and ambulatory arterial stiffness index (AASI).

#### Cognitive assessment

All cognitive assessments were conducted by the same examiner, specifically dedicated to the project and trained in the administration of the study tests. Evaluations were performed at baseline, prior to initiation of the dietary supplement, and repeated at 6 and 12 months.

The following tests were administered: *Montreal Cognitive Assessment (MoCA)*, cognitive screening tool, adjusted for age and educational level; Trail Making Test (TMT) A and B: assessing attention, psychomotor speed and cognitive flexibility; Symbol Digit Modalities Test (SDMT): evaluating processing speed, visual perception, stimulus recognition, attention, task supervision and interference control; Five Digit Test: measuring cognitive processing speed as well as specific aspects of attention and executive function.

#### Physical function assessment

Physical function assessment included the evaluation of gait speed, walking endurance and balance as indicators of lower limb function, as well as upper limb strength. Gait speed was assessed with the GAITRite system (CIR Systems Inc., Franklin, NJ, USA),^13^ an instrumented walkway system for gait analysis that provides spatiotemporal gait parameters, while walking endurance was determined using the Six-Minute Walking Test (6MWT).^14^ Gait analysis was performed before and after the 6MWT to examine the impact of sustained exertion on gait speed.^15^ Balance was evaluated using the Berg Balance Scale, which assesses static and dynamic balance through 14 items.^16^ Finally, the upper limb strength, assessed as handgrip strength, was quantified using a manual dynamometer (Smedley, Takei Scientific Instruments Co., Ltd., Niigata, Japan).^17^

All measurements were conducted by a physiotherapist researcher blinded to group allocation.

#### Neuroimaging

Magnetic resonance imaging examinations were performed using a Philips Ingenia 3.0 Tesla device available at the Neuroradiology Department of Virgen del Rocío University Hospital (Seville, Spain), dedicated to research purposes. The study protocol was designed to maximise the detection of ischaemic lesions at any brain location and included a specific sequence for the assessment of parenchymal microbleeds.

Each MRI session consisted of 5 sequences:

- Localisation sequence: Non-diagnostic; used solely for planning subsequent scans.
- 3D T1 Turbo Field Echo (TFE): High spatial resolution sequence providing detailed anatomical information and allowing multiplanar assessment.
- 3D T2 Fluid-Attenuated Inversion Recovery (FLAIR): High spatial resolution sequence with strong diagnostic capacity, also allowing multiplanar evaluation.
- Diffusion-weighted imaging (DWI): Axial 2D sequence, 5 mm slice thickness, with maximum sensitivity for detecting acute and subacute infarcts, including 3 image series: b0, b1000 and the apparent diffusion coefficient map.
- Susceptibility-weighted imaging: Axial 2D sequence, 3 mm slice thickness, that enhances paramagnetic artefacts from haemosiderin deposits, enabling the detection of microbleeds.

**Radiological variables:** Brain MRI scans were all read by an experienced neuroradiologist enrolled in the project. Radiological features related with SVD were described following the 2023 STRIVE group (STRIVE-2) neuroimaging standards for research into SVD^18^: absence/presence, number and location of recent subcortical infarcts (acute/subacute phase was further assessed according to DWI behaviour); absence/presence, number and location of ischaemic lacunar lesions; absence/presence, number and location of haemorrhagic lacunar lesions; absence/presence, number (<10, 10–20, > 20) and location of brain microbleeds; absence/presence and global distribution of dilated perivascular spaces; degree of FLAIR hyperintensities of presumed vascular origin according to the Fazekas scales (subcortical, periventricular and total); absence/presence of superficial cortical siderosis and absence/presence and location of cortical microinfarcts. The absence/presence and location of territorial infarcts and major haemorrhagic events was also registered.

**Management of incidental findings:** All pathological findings not related to cerebrovascular disease were reported.

### Statistical analysis

Data were collected using the Research Electronic Data Capture (REDCap) platform. Safety analyses were performed according to the intention-to-treat principle, including all participants who initiated treatment. Statistical analyses were conducted using SPSS (version 29) and R (version 4.3.1). As this was an exploratory pilot study primarily designed to evaluate safety and feasibility, no formal power calculation for efficacy outcomes was performed. The sample size was determined based on feasibility considerations, including extract production capacity and expected recruitment rate.

Descriptive statistics were used to characterise the sample. Qualitative variables were summarised as absolute and relative frequencies, while quantitative variables were described using measures of central tendency and dispersion. Normality of quantitative variables was assessed with the Shapiro–Wilk test. Based on this assessment, independent-sample Student’s *t*-tests were applied for normally distributed variables, or Mann–Whitney *U* tests in the case of non-normality. Associations between qualitative variables were analysed using the chi-square test.

Because baseline imbalances were observed between groups (age, hypertension and smoking status), all statistical models were adjusted for age, hypertension, smoking status and baseline values of the outcome variable. Mixed linear models and generalised linear mixed models were employed depending on the data structure and the distribution of dependent variables. In generalised models, Gamma distributions with a log link were primarily used; when necessary, lognormal or T-family distributions were additionally applied to improve model fit and simulation of observed data.

Model validation was performed through residual analyses, graphical evaluations and specific goodness-of-fit tests, including methods based on simulated residuals and verification of assumptions for random effects. For comparisons between groups and within groups over time (baseline, mid-study and final visits), multiplicity corrections were applied using the Bonferroni method.

A *P*-value < .05 was considered statistically significant.

## Results

### Participants and safety evaluation

**Figure 1:**
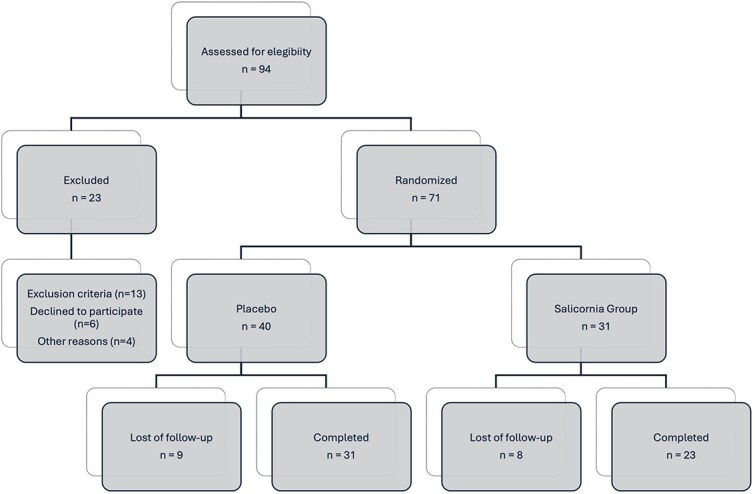
Flow diagram of the study.

A total of 71 patients were randomised in the study, with 40 allocated to the placebo group and 31 to the *Salicornia* extract group. The participant flow through the trial is summarised in Figure 1.

At baseline, patients in the placebo group were older than those in the *Salicornia* group (median 69 vs 64 years, *P* = .032). The mean time from the index stroke to randomisation was 12.6 months (range 1–24 months), with no significant differences between groups. Significant differences were also observed in smoking status (*P* = .037) and hypertension prevalence (93% vs 68%, *P* = .018), while other cardiovascular risk factors were similarly distributed between groups. Details are shown in Table 1.

**Table 1:** Baseline characteristic.

	Overall (*n* = 71)	Placebo (*n* = 40)	*Salicornia* extract (*n* = 31)	*P*
**Age (median [min; max])**	65 (58; 71)	69 (60; 77)	64 (57; 67)	**.032**
**Male/female (*n*)**	53/18	30/10	23/8	.938
**Tobacco used (*n*, %)**				**.037**
**No**	28 (39%)	21 (53%)	7 (23%)	
**Yes**	24 (34%)	11 (28%)	13 (42%)	
**Former**	19 (27%)	8 (20%)	11 (35%)	
**Alcohol (*n*, %)**				.326
**No**	56 (79%)	31 (78%)	25 (81%)	
**Yes**	9 (13%)	4 (10%)	5 (16%)	
**Former**	6 (8.5%)	5 (13%)	1 (3.2%)	
**Hypertension (*n*, %)**	58 (82%)	37 (93%)	21 (68%)	**.018**
**Diabetes (*n*, %)**	27 (38%)	15 (38%)	12 (39%)	.917
**Dyslipidaemia (*n*, %)**	42 (60%)	25 (64%)	17 (55%)	.663
**Atrial fibrillation (*n*, %)**	6 (8.5%)	5 (13%)	1 (3.2%)	.363
**Prior stroke/transient ischaemic attack (*n*, %)**	7 (9.9%)	4 (10%)	3 (9.7%)	.964
**Peripheral vascular disease (*n*, %)**	6 (8.5%)	3 (7.5%)	3 (9.7%)	.944
**Coronary disease (*n*, %)**	10 (14%)	5 (13%)	5 (16%)	.963
**Withdrawal (*n*, %)**	17 (24%)	9 (23%)	8 (26%)	.976
**Adverse event (*n*, %)**	25 (35%)	17 (43%)	8 (26%)	.234

Boldface used to highlight values considered clinically or statistically relevant.

Safety analyses were performed according to the intention-to-treat principle. Overall, 17 patients (24%) withdrew from the study, with similar rates between the placebo (23%) and *Salicornia* groups (26%, *P* = .976).

Safety outcomes were systematically monitored throughout the study through scheduled visits and telephone follow-up. Adverse events were documented and classified according to severity and potential relationship with the intervention. Most reported events were mild and self-limited. Four participants discontinued the study because of AEs, all of which were gastrointestinal in nature; 3 occurred in the placebo group and 1 in the *Salicornia* group. No serious AEs were considered related to the investigational product.

The distribution of AEs between groups is shown in Table 1. Importantly, no recurrent ischaemic stroke, transient ischaemic attack or myocardial infarction was observed during follow-up.

To explore the potential impact of attrition on study outcomes, baseline characteristics of participants who completed follow-up and those who withdrew were compared. No significant differences were observed in baseline cognitive scores or other major clinical variables between completers and non-completers, although this comparison does not exclude the possibility that missing follow-up data influenced the interpretation of cognitive outcomes.

Adherence to the intervention was estimated from capsule return counts at each follow-up period, assuming 360 capsules dispensed per 6-month period. For descriptive purposes, participants with capsule-count data available were classified as having adherence ≥ 80% or < 80%; participants without capsule-count data at the corresponding visit were considered unevaluable for this analysis. No participant had capsule counts compatible with complete non-use of the dispensed treatment. At 6 months, adherence ≥ 80% was observed in 33/34 (97.1%) placebo-treated and 24/25 (96.0%) *Salicornia*-treated participants with evaluable capsule-count data. At 12 months, the corresponding proportions were 29/30 (96.7%) and 19/20 (95.0%), respectively. Capsule-count data were unavailable for 6/40 placebo-treated and 6/31 *Salicornia*-treated participants at 6 months, and for 10/40 and 11/31, respectively, at 12 months. These adherence estimates should therefore not be interpreted as a direct measure of long-term tolerability in the whole randomised sample.

### Efficacy evaluation

#### Analytical evaluation

To assess systemic effects of *Salicornia* supplementation, fasting blood samples were analysed at baseline and during follow-up visits. No significant differences were observed between groups in glucose, creatinine, glomerular filtration rate, albumin, sodium, TSH, HbA1c, CRP, vitamin B12, folic acid or lipid profile at any study visits.

Potassium levels were consistently higher in the *Salicornia* group compared with the placebo group at all time points, reaching statistical significance at visits 1, 2 and 3 (*P* = .019, *P* = .009 and *P* = .022, respectively).

Homocysteine concentrations decreased significantly from baseline to follow-up in both groups (*P* < .01). Participants receiving *Salicornia* exhibited a lower mean homocysteine level, although the between-group difference did not reach statistical significance.

Total cholesterol, HDL, LDL and triglyceride levels remained stable throughout the study, with no significant differences between groups. Details are shown in Table 2.

**Table 2:** Analytical data.

	Placebo *n* = 40	*Salicornia* extract *n* = 31	*P*	Placebo *n* = 36	*Salicornia* extract *n* = 28	*P*	Placebo *n* = 31	*Salicornia* extract *n* = 23	*P*
	**Visit 1**			**Visit 2**			**Visit 3**		
**Glucose**	103.68 (96.09–112.00)	106.12 (98.48–114.49)	.644	110.78 (102.31–120.10)	112.46 (103.93–121.84)	.777	102.09 (94.15–110.88)	105.92 (97.53–115.21)	.505
**Creatinine**	0.79 (0.79–0.89)	0.84 (0.76–0.93)	.434	0.81 (0.73–0.90)	0.81 (0.73–0.89)	.969	0.81 (0.73–0.90)	0.83 (0.75–0.92)	.706
**eGFR**	87.38 (81.89–93.24)	84.36 (78.70–90.42)	.439	84.84 (79.41–90.42)	85.62 (79.79–91.88)	.843	86.05 (80.39–92.11)	83.71 (77.82–90.04)	.565
**Albumin**	4.54 (4.43–4.65)	4.54 (4.43–4.65)	.994	4.45 (4.34–4.57)	4.56 (4.45–4.67)	.148	4.57 (4.46–4.69)	4.56 (4.44–4.68)	.882
**Sodium**	141.06 (140.20–141.92)	141.19 (140.33–142.06)	.818	**139.87 (139.98–140.77)‡**	140.93 (140.02–141.83)	.082	140.63 (139.69–141.57)	140.59 (139.62–141.57)	.955
**Potassium**	**4.27 (4.10–4.44)**	**4.54 (4.37–4.71)**	**.019**	**4.29 (4.12–4.48)**	**4.61 (4.43–4.79)**	**.009**	**4.20 (4.01–4.39)**	**4.49 (4.29–4.68)**	**.022**
**TSH**	1.48 (1.19–1.85)	1.26 (1.02–1.55)	.234	1.65 (1.32–2.06)	**1.50 (1.22–1.86)**‡	.514	1.45 (1.16–1.82)	1.49 (1.19–1.85)	.882
**HbA1C**	6.08 (5.81–6.37)	6.29 (6.02–6.58)	.237	6.13 (5.86–6.43)	6.04 (5.77–6.34)	.277	6.04 (5.77–6.34)	6.21 (5.94–6.51)	.359
**CRP**	2.14 (1.62–2.83)	2.06 (1.52–2.79)	.851	1.69 (1.27–2.25)	2.30 (1.69–3.15)	.156	2.09 (1.55–2.83)	1.88 (1.36–2.62)	.646
**Vitamin B12 (pg/mL)**	345.83 (291.20–410.69)	350.22 (296.78–413.29)	.909	320.08 (267.95–382.36)	334.80 (281.68–397.95)	.699	345.67 (287.04–416.26)	346.36 (288.41–415.96)	.987
**Folate (ng/mL)**	6.24 (5.31–7.33)	5.74 (4.92–6.69)	.424	6.67 (5.64–7.88)	6.54 (5.57–7.67)	.853	6.62 (5.57–7.88)	5.89 (4.98–6.97)	.302
**Homocysteine (μmol/L)**	15.29 (13.49–17.33)	14.39 (12.78–16.19)	.444	**12.89 (11.34–14.65)^†^**	**11.68 (10.33–13.18)^†^**	.229	**12.82 (11.24–14.61)^†^**	**12.16 (10.72–13.81)^†^**	.538
**Total cholesterol**	147.15 (131.47–164.70)	143.89 (129.91–159.38)	.729	148.72 (132.60–166.79)	144.11 (129.73–160.08)	.636	149.70 (133.12–168.36)	136.54 (122.44–152.28)	.183
**HDL**	48.38 (42.92–54.54)	49.28 (44.29–54.83)	.782	48.38 (42.87–54.61)	46.84 (42.04–52.19)	.635	47.80 (42.31–54.01)	**45.44 (40.69–54.73)^‡^**	.464
**LDL**	72.76 (61.85–85.59)	68.96 (59.38–80.08)	.569	74.12 (62.71–87.61)	74.69 (64.17–86.94	.937	77.65 (65.55–91.99)	67.95 (58.02–79.56)	.183
**Triglycerides**	109.65 (90.93–132.21)	103.98 (87.06–124.18)	.655	111.48 (92.16–134.84)	98.35 (82.03–117.92)	.302	112.77 (92.84–136.97)	103.67 (85.98–124.99)	.501

Boldface used to highlight values considered clinically or statistically relevant. Abbreviations: CRP, C-reactive protein; eGFR, estimated glomerular filtration rate; HbA1c, glycated haemoglobin; HDL, high-density lipoprotein; LDL, low-density lipoprotein; TSH, thyroid-stimulating hormone.

^†^ *P* < .001 (relative to visit 1).

^‡^ *P* < .05 (relative to visit 1).

#### Ambulatory blood pressure monitoring

Ambulatory blood pressure monitoring showed no significant differences between groups in average SBP, DBP, MAP, heart rate or pulse pressure at any visit. At the end of follow-up (visit 3), SBP and DBP values were similar between groups (placebo: 128.86/75.6 mmHg vs *Salicornia*: 125.38/70.9 mmHg), with no statistically significant differences observed. In the placebo group, blood pressure values remained relatively stable over time. In the *Salicornia* group, a trend towards lower DBP was observed at visit 3 compared with placebo (70.9 vs 75.6 mmHg, *P* = .053), although this did not reach statistical significance. Morning surge index and AASI showed no relevant differences between groups during follow-up.

#### Cognitive assessment

Cognitive performance was evaluated at baseline and during follow-up using standardised neuropsychological tests. Baseline cognitive values are shown in Table 3. At follow-up, cognitive results are presented as estimated marginal means derived from the fully adjusted models. At 12 months, MoCA data were available for 22/40 participants in the placebo group and 19/31 participants in the *Salicornia* group, corresponding to missing data rates of 45% and 38%, respectively. Among participants with available 12-month follow-up data, adjusted MoCA scores were higher in the *Salicornia* group than in the placebo group.

**Table 3:** Cognitive assessment—models adjusted for age, hypertension, smoking status and baseline values.

	Placebo (*n* = 40)	*Salicornia* extract (*n* = 31)	*P*	Placebo *n* = 23	*Salicornia* extract *n* = 26	*P*	Placebo *n* = 22	*Salicornia n* = 19	*P*
	**Visit 1**			**Visit 2**			**Visit 3**		
**MoCA—direct score**	21.73 (19.67–23.79)	22.89 (21.38–24.41)	.271	22.96 (21.32–24.60)	24.54 (23.28–25.79)	.082	23.93 (22.25–25.61)	25.36 (23.80–26.93)	.142
**MoCA—adjusted (age and education)**	7.69 (6.18–9.61)	7.96 (6.68–9.48)	.779	8.91 (7.02–10.79)	10.22 (8.81–11.63)	.193	**9.60 (7.68–11.51)**	**11.82 (10.10–13.54)**	**.042**
**SDMT**	10.01 (8.74–11.29)	9.31 (8.28–10.36)	.321	9.93 (8.57–11.28)	10.59 (9.57–11.59)	.361	10.77 (9.37–12.18)	10.31 (9.17–11.45)	.549
**5DIG—flexibility**	2.30 (0.05–7.87)	10.71 (4.75–19.05)	.017	**10.50 (1.41–28.05)**	**34.56 (20.20–52.76)**	**.013**	**36.52 (15.88–65.63)†**	36.58 (19.36–59.22)	.997
**5DIG—inhibition**	31.43 (15.91–46.96)	25.68 (12.88–38.46)	.504	47.41 (27.46–67.35)	37.29 (22.18–52.40)	.314	48.41 (27.93–68.89)	41.68 (24.89–58.47)	.534
**TMT-A**	11.02 (9.30–12.75)	10.63 (9.25–12.02)	.672	11.33 (9.67–12.99)	11.35 (9.98–12.73)	.983	11.21 (9.43–12.99)	10.58 (9.09–12.08)	.529
**TMT-B**	9.92 (8.40–11.44)	10.05 (8.76–11.34)	.873	10.07 (8.05–12.10)	10.71 (9.19–12.23)	.530	10.85 (8.69–13.01)	**9.04 (7.35–10.74)**‡	.117

Boldface used to highlight values considered clinically or statistically relevant. Abbreviations: 5DIG, Five Digit Test; EMMs, estimated marginal means; MoCA, Montreal Cognitive Assessment; SDMT, Symbol Digit Modality Test; TMT-A, Trail Making Test, Part A; TMT-B, Trail Making Test, Part B.

Baseline values (visit 1) are shown descriptively. Values at visits 2 and 3 represent EMMs with 95% CIs derived from the adjusted models. *P* values correspond to between-group comparisons at each visit. Symbols (†, ‡) indicate within-group changes relative to baseline. The row “MoCA-adjusted (age and education)” shows the age- and education-adjusted MoCA scores.

In the adjusted analyses accounting for age, hypertension, smoking status and baseline values, some cognitive measures were higher in the *Salicornia* group during follow-up. Direct MoCA scores were numerically higher in the *Salicornia* group at visits 2 and 3 compared with the placebo group, although these differences did not reach statistical significance in the fully adjusted models. The age- and education-adjusted MoCA values, shown in the row “MoCA-adjusted (age and education)” in Table 3, showed a significant between-group difference at visit 3 (*P* = .042).

To improve clinical interpretation, we performed an additional descriptive analysis of clinically meaningful change in MoCA score among participants with baseline and 12-month data available; however, this analysis should be interpreted cautiously given the small numbers and missing follow-up data. An improvement of ≥ 2 points was observed in 45.5% of placebo-treated participants (10/22) and 58.8% of *Salicornia*-treated participants (10/17). In addition, crossing from MoCA < 26 at baseline to MoCA ≥ 26 at 12 months was observed in 18.2% of the placebo group (4/22) and 47.1% of the *Salicornia* group (8/17).

Regarding executive function, the Five Digit flexibility subtest showed higher scores in the *Salicornia* group at visit 2 (*P* = .013), although this difference was not maintained at visit 3. Other cognitive measures, including TMT-A, TMT-B, SDMT and the Five Digit inhibition subtest, did not show significant between-group differences in the adjusted analyses, although a trend towards improvement in TMT-B was observed at visit 3 in the *Salicornia* group.

#### Physical function assessment

Regarding lower limb function, baseline gait speed was comparable between groups. In the adjusted analyses accounting for age, hypertension, smoking status and baseline values, patients receiving *Salicornia* supplementation had higher gait speed during follow-up than the placebo group in the adjusted analyses. Gait speed was consistently higher in the *Salicornia* group during follow-up. At visit 2, the adjusted pre-test gait speed was 121.32 cm/s in the *Salicornia* group compared with 109.21 cm/s in the placebo group, corresponding to an adjusted mean difference of 12.11 cm/s (95% CI, 3.87–20.35; *P* = .004).

In contrast, walking distance showed a clear between-group difference during follow-up. Patients receiving *Salicornia* supplementation achieved greater walking distances compared with placebo during follow-up. At visit 2, the adjusted mean distance was 505.94 m in the *Salicornia* group vs 391.91 m in the placebo group, corresponding to an adjusted mean difference of 114.03 m (95% CI, 53.63–174.43; *P* < .001). These findings remained consistent in the fully adjusted models. All results are presented in Table 4.

**Table 4:** Gait analysis—models adjusted for age, hypertension, smoking status and baseline values.

	Placebo*n* = 40	*Salicornia* extract *n* = 31	*P*	Placebo*n* = 34	*Salicornia* extract *n* = 23	*P*	Placebo*n* = 29	*Salicornia* extract *n* = 22	*P*
	**Visit 1**			**Visit 2**			**Visit 3**		
**Speed (cm/s)—pre**	113.92 (103.16–124.69)	105.82 (96.35–115.29)	.194	**109.21 (101.82–116.60)**	**121.32 (115.07–127.57)**	**.004**	**111.83 (104.15–119.50)^‡^**	**123.74 (117.29–130.19)†**	**.006**
**Speed (cm/s)—post**	**127.46 (115.56–140.59)**	**113.26 (104.12–123.20)**	**.037**	115.16 (104.47–125.84)	127.19 (118.22–136.16)	.053	118.83 (108.22–129.65)	127.85 (118.47–137.23)	.153
**Distance (m)**	428.28 (383.10–473.47)	408.97 (367.73–450.20)	.446	**391.91 (356.29–427.49)**	**505.94 (475.10–536.77)**	**<.001**	**408.45 (372.11–444.80)**	**464.28 (432.28–496.29)** ^‡^	**.008**
**Handgrip strength (kg)—right**	26.47 (23.24–29.70)	26.40 (22.38–30.43)	.981	**22.02 (18.92–25.12)**	**28.35 (25.23–31.47)**	**.002**	**27.05 (23.86–30.24)^†^**	29.54 (26.40–32.68)	.218
**Handgrip strength (kg)—left**	25.95 (21.99–29.91)	24.20 (20.31–28.10)	.339	24.25 (21.29–27.22)	26.50 (23.73–29.26)	.229	**28.88 (25.91–31.86)^†^**	26.25 (23.41–29.07)	.160
**Berg Balance Scale**	54.78 (51.15–58.41)	51.74 (48.59–54.89)	.139	52.94 (50.59–55.29)	54.72 (52.70–56.74)	.187	52.18 (49.77–54.58)	54.31 (52.23–56.39)	.663

Baseline values (visit 1) are shown descriptively. Values at visits 2 and 3 represent estimated marginal means (EMMs) with 95% CIs derived from the adjusted models. *P* values correspond to between-group comparisons at each visit. Symbols (†, ‡) indicate within-group changes relative to baseline. Boldface used to highlight values considered clinically or statistically relevant.

Balance performance, assessed using the Berg Balance Scale, showed modest improvements during follow-up in the *Salicornia* group. However, in the adjusted models accounting for age, hypertension, smoking status and baseline values, no statistically significant between-group differences were observed at follow-up visits. Regarding upper limb strength, handgrip strength showed a transient increase on the right side at visit 2 in the *Salicornia* group, although this finding was not consistent across time points or sides and did not translate into sustained between-group differences in the adjusted analyses.

#### Neuroimaging

Neuroimaging analyses performed at baseline and at study completion showed no significant between-group differences in the incidence of new infarcts. At the start of follow-up, the placebo group exhibited higher odds of presenting a haemorrhagic lacunar lesion (OR = 7.84; 95% CI, 1.59–38.75; *P* = .0115). Although this difference persisted at the end of follow-up (OR = 5.14; 95% CI, 0.95–27.75), it did not reach statistical significance (*P* = .057). By the end of the study, no relevant between-group differences were observed in the occurrence of ischaemic lesions, perivascular space burden, cerebral microbleeds or in the severity of white-matter hyperintensities as assessed by the Fazekas scales.

## Discussion

In this randomised, double-blind pilot trial, supplementation with a polyphenol-rich *S ramosissima* extract showed no treatment-related serious AEs in patients with lacunar stroke. However, interpretation of the exploratory differences observed in some cognitive and physical outcomes is substantially limited by the pilot nature of the study, the baseline imbalance between groups in key prognostic variables and the high proportion of missing follow-up data, particularly for cognitive measures. Therefore, these findings should be considered hypothesis-generating only and should not be interpreted as evidence of confirmed efficacy.

The study incorporated a comprehensive multimodal assessment including cognitive testing, gait analysis, biochemical biomarkers and neuroimaging. This design provides preliminary information on the safety of long-term supplementation and on possible clinical signals that may warrant evaluation in larger, adequately powered studies, although any apparent differences in secondary exploratory outcomes must be interpreted cautiously.

From a biochemical perspective, we observed a reduction in homocysteine levels, consistent with previous work from our group demonstrating the homocysteine-lowering properties of this extract.^10^ Polyphenols are known to modulate the oxidative and inflammatory cascades, which may counteract mechanisms triggered by elevated homocysteine levels. In addition, they may influence enzymatic pathways involved in its metabolism, potentially contributing to improved vascular and neuronal homeostasis. Furthermore, polyphenol-rich dietary patterns such as the Mediterranean diet have been associated with lower homocysteine concentrations and reduced incidence of neurovascular disorders.^19^ The link between hyperhomocysteinemia, vascular cognitive impairment and stroke recurrence is well established,^20^ which supports the biological plausibility of this observation. However, because homocysteine levels also decreased in the control group, it cannot be excluded that unreported treatments, dietary changes or lifestyle modifications may have contributed to these changes.

Polyphenols have additional recognised vascular benefits, including inhibition of platelet aggregation, attenuation of LDL oxidation, improvement of endothelial function and modulation of antioxidant and anti-inflammatory responses.^21^ In our study, however, no clear effects on LDL levels or blood pressure were observed, beyond a trend towards lower diastolic pressure in the treatment group. These findings may partly reflect baseline imbalances between groups, as participants receiving *Salicornia* were older and more frequently hypertensive, while vascular risk factors were largely well controlled at baseline. Such conditions may produce a ceiling effect that limits the detection of additional improvements. Furthermore, concomitant lipid-lowering therapy could have masked changes in LDL, contrasting with our prior observations in healthy untreated volunteers, where lipid reductions were more evident.^10^ Although several neurovascular and neuroprotective mechanisms have been proposed for polyphenols, these were not directly assessed in the present study. Therefore, the mechanistic interpretations discussed here should be considered biologically plausible but speculative. This cautious interpretation is further supported by the absence of significant between-group differences in LDL cholesterol, blood pressure and neuroimaging markers, together with the reduction in homocysteine observed in both groups.

Regarding neuropsychological outcomes, exploratory differences in MoCA scores were observed in the treatment group during follow-up. Although these descriptive findings may support the potential clinical relevance of the observed MoCA changes, they should be interpreted cautiously given the small sample size, missing follow-up data and the possibility of practice effects related to repeated cognitive testing. The MoCA is a sensitive screening tool for mild vascular cognitive impairment and is well suited for patients with SVD-related deficits.^22^ Vascular cognitive impairment is prevalent in lacunar stroke, affecting up to 40% of patients and is characterised primarily by executive dysfunction and reduced processing speed. Established risk factors include diabetes, depressive symptoms, elevated BMI and greater white matter hyperintensity (WMH) burden.^23^ While intensive blood pressure control and multidomain lifestyle interventions have shown some benefit, there are still no disease-modifying pharmacological therapies. Consistent with prior randomised evidence, polyphenol-rich nutraceuticals have been associated with improvements in executive function and episodic memory in older adults,^24^ primarily through the reduction of oxidative stress and the regulation of signalling pathways associated with synaptic plasticity.^25^ Our findings may be compatible with a possible role of polyphenol-based nutraceutical strategies in cognitive improvement,^26,27^ although this interpretation remains highly uncertain given the exploratory nature of the analysis, the missing follow-up data and the possibility of practice effects related to repeated cognitive testing. Nevertheless, clinical evidence in humans remains limited, and further mechanistic exploration is warranted.

Gait impairment is another hallmark feature of SVD. Multiple MRI markers, including lacunes, WMH, cerebral microbleeds, atrophy and reduced white matter integrity have been linked to disturbances in gait and mobility. Importantly, gait abnormalities are predictors of cognitive decline and dementia in SVD.^2^ In our trial, apparent differences in gait speed and distance may suggest possible effects of polyphenol supplementation, although these findings remain exploratory and should be interpreted cautiously. Polyphenols have been shown to enhance muscle strength and walking speed in other settings,^28^ which may support this possibility.

Neuroimaging analyses did not show significant differences between groups in new ischaemic lesions, perivascular space burden, cerebral microbleeds or WMH severity at study completion. Given the slow progression of cSVD and the 1-year duration of the trial, the absence of structural MRI changes is not unexpected. These findings are consistent with longitudinal neuroimaging evidence showing that most cSVD markers, particularly white matter hyperintensities, lacunes and cerebral microbleeds progress over time, with faster progression observed in older individuals.^29^

A major strength of this trial is its rigorous randomised, double-blind, placebo-controlled design, which minimises bias and enhances the validity of observed clinical effects. The comprehensive multimodal assessment, including standardised cognitive batteries sensitive to vascular cognitive impairment and objective gait and balance measures, provides a broad evaluation of functional outcomes relevant to patients with lacunar stroke.

Several limitations should nevertheless be acknowledged. First, the relatively small sample size and pilot nature of the study, which was primarily aimed at evaluating safety and feasibility, mean that the analyses of clinical outcomes should be considered exploratory and hypothesis-generating rather than confirmatory. Second, baseline imbalances between groups likely reflect the use of simple randomisation in a relatively small sample and may have influenced exploratory outcomes despite statistical adjustment. Third, the large number of exploratory endpoints increases the risk of type I error, and therefore, the observed associations should be interpreted with caution. The bioavailability and metabolism of distinct polyphenolic compounds in the *Salicornia* extract were not directly measured, and variations in these parameters can critically influence central and systemic effects. In addition, the withdrawal rate may have reduced the effective statistical power of the study and represents a potential source of attrition bias, although no significant baseline differences were identified between completers and non-completers. This concern is particularly relevant for cognitive outcomes, for which 12-month MoCA data were available for only 22/40 placebo-treated and 19/31 *Salicornia*-treated participants. Finally, dietary habits, concomitant medications and other lifestyle factors, which are common challenges in nutritional intervention studies, may also have influenced the observed outcomes. Detailed information on concomitant pharmacological therapies and non-pharmacological interventions, such as rehabilitation programs or dietary modifications, was not systematically collected for formal between-group comparison and may therefore have acted as residual confounders.

## Conclusions

In summary, this pilot trial suggests that long-term supplementation with a polyphenol-rich *S ramosissima* extract had an acceptable safety profile in patients with lacunar stroke, although long-term tolerability should be interpreted cautiously in view of the withdrawal rate. Any apparent differences in secondary exploratory cognitive, functional and biochemical outcomes should be interpreted with caution in view of the baseline imbalances, small sample size and substantial missing follow-up data. Larger, adequately powered trials are needed to determine whether these preliminary findings represent true treatment effects.

## Supplementary Material

aakag049_Supplemental_Files

## Data Availability

The present study is compliant with the journal’s data availability standards, and any data not provided in the article may be shared by request of other qualified investigators. The authors have full access to all the data in the study and take responsibility for its integrity and data analysis.

## Clinical trial registration

NCT06076122.
