# Supplementary material for: Polyphenol-rich Salicornia extract in lacunar stroke: a pilot randomised trial of safety and exploratory clinical outcomes
Source: Eur Stroke J. 2026 May 20;11(5):aakag049. doi: 10.1093/esj/aakag049 (PMC13189269; doi:10.1093/esj/aakag049)
Supplement: aakag049_Supplemental_Files [file aakag049_supplemental_files.zip › Supplementary_Files_Adjusted_Models_aakag049.docx]

Supplementary Files

Gait Analysis – Models adjusted for age

|  | **PLACEBO** | **SALICORNIA EXTRACT** | ***P*** | **PLACEBO**  **N=34** | **SALICORNIA EXTRACT**  **N=23** | ***P*** | **PLACEBO**  **N=29** | **SALICORNIA EXTRACT**  **N=22** | ***P*** |
| --- | --- | --- | --- | --- | --- | --- | --- | --- | --- |
|  | **VISIT 1** | | | **VISIT 2** | | | **VISIT 3** | | |
| **SPEED (meters per second m/s) - pre** | 112.61 (103.63-121.60) | 105.36 (97.77-114.95) | 0.232 | 117.36 (108.13-126.59) | **124.54 (108.13-134.26)** † | 0.247 | **122.45 (112.95-131.95)** ‡ | **127.84 (117.98-137.71)** † | 0.396 |
| **SPEED (m/s) - post** | **125.26 (115.54-135.79)** | **112.71 (103.78-122.41)** | **0.045** | 125.95 (115.93-136.83) | **128.35 (118.36-139.18)** † | 0.721 | 126.09 (116.01-137.04) | **126.36 (116.39-137.19)** † | 0.968 |
| **DISTANCE (meters)** | 419 (381.84-456.24) | 403.39 (363.39-443.39) | 0.539 | **416.46 (377.86-455.06)** | **510.94 (470.45-551.42)** † | <0.001 | 432.14 (392.80-471.48) | **478.38 (437.04-519.73)** † | 0.083 |
| **DINAMO (kilograms, kg) - RIGHT** | 26.47 (23.24-29.70) | 26.40 (22.38-30.43) | 0.981 | **21.59 (18.25-24.94)** | **27.66 (23.57-31.75)** | **0.026** | **26.31 (22.88-29.74)** | 28.58 (24.46-32.69) | 0.407 |
| **DINAMO (kg) - LEFT** | 25.99 (22.94-29.06) | 23.61 (19.79-27.43) | 0.339 | 22.05 (18.90-25.21) | 25.12 (21.24-28.99) | 0.231 | 27.12 (23.87-30.36) | 24.52 (20.62-28.42) | 0.316 |
| **BERG scale** | 55 (51.77-56) | 54 (51-56) | 0.389 | **54.5 (52-56)** | **56 (55-56)** | **0.008** | **54 (52-56)** | **56 (54.2-56)** | **0.013** |

*Values represent adjusted estimated means (95% CI). P values correspond to between-group comparisons at each visit derived from the adjusted models. Symbols (†, ‡) indicate within-group changes relative to baseline.*

*† p<0.001 (relative to visit 1). ‡ p<0.05 (relative to visit 1)*

Cognitive Assessment – Models adjusted for age

|  | **PLACEBO** | **SALICORNIA EXTRACT** | ***P*** | **PLACEBO**  **N=23** | **SALICORNIA EXTRACT**  N=26 | ***P*** | **PLACEBO**  N=22 | **SALICORNIA**  **EXTRACT**  N=19 | ***P*** |
| --- | --- | --- | --- | --- | --- | --- | --- | --- | --- |
|  | **VISIT 1** | | | **VISIT 2** | | | **VISIT 3** | | |
| **Montreal Cognitive Assessment (MOCA) - Direct Score** | 22.50 (20.77-24.24) | 23.21 (21.77-24.65) | 0.195 | **22.49 (20.65-24.34)** | **24.91 (23.47-26.36)** † | **0.023** | **23.67 (21.81-25.54)** | **26.01 (24.46-27.55)** † | **0.036** |
| **MOCA - Adjusted**  **(age and education)** | 8.21 (6.85-9.84) | 8.17 (6.92-9.65) | 0.967 | 8.26 (6.82-10.00) | **9.79 (8.29-11.57)** ‡ | 0.141 | 9.03 (7.44-10.96) | **10.94 (9.13-13.10)** † | 0.116 |
| **(Symbol Digit Modality Test (SDMT)** | 11.53 (9.46-11.60) | 9.54 (8.54-10.54) | 0.140 | 10.50 (9.33-11.68) | **10.57 (9.55-11.59)** | 0.929 | 11.35 (10.12-12.57) | **10.54 (9.42-11.65)** | 0.298 |
| **Five digit Test (5DIG)-flexibility** | 6.69 (2.11-13.85) | 13.44 (6.57-22.75) | 0.143 | **12.41 (4.83-23.50)** | **36.96 (24.94-51.35)** † | **0.001** | **34.66 (20.57-52.42)** † | **37.01 (23.63-53.39)** † | 0.819 |
| **Five digit Test (5DIG) -inhibition** | 36.07 (22.75-49.39) | 26.59 (13.81-39.38) | 0.259 | 46.09 (30.54-61.65) | 28.85 (15.76-41.94) | 0.069 | 43.76 (27.67-59.85) | 32.42 (17.92-46.92) | 0.266 |
| **Trail Making Test part A (TMT-A)** | 11.05 (9.63-12.46) | 10.63 (9.33-11.94) | 0.639 | 11.78 (10.25-13.30) | 11.57 (10.20-12.93) | 0.824 | 11.73 (10.12-13.34) | 11.11 (9.68-12.54) | 0.534 |
| **Trail Making Test, part B (TMT-B)** | 10.03 (8.80-11.25) | 10.08 (8.86-11.29) | 0.947 | 9.93 (8.59-11.27) | 10.60 (9.39-11.80) | 0.420 | 10.52 (9.01-12.02) | 8.96 (7.64-10.28) | 0.104 |

*Values represent adjusted estimated means (95% CI). P values correspond to between-group comparisons at each visit derived from the adjusted models. Symbols (†, ‡) indicate within-group changes relative to baseline.*

*† p<0.001 (relative to visit 1). ‡ p<0.05 (relative to visit 1)*

Gait analysis – Models adjusted for age, hypertension, smoking status, and baseline Values

|  | **PLACEBO** | **SALICORNIA EXTRACT** | ***P*** | **PLACEBO**  **N=34** | **SALICORNIA EXTRACT**  **N=23** | ***P*** | **PLACEBO**  **N=29** | **SALICORNIA EXTRACT**  **N=22** | ***P*** |
| --- | --- | --- | --- | --- | --- | --- | --- | --- | --- |
|  | **VISIT 1** | | | **VISIT 2** | | | **VISIT 3** | | |
| **SPEED (meters per second m/s) - pre** | 113.92 (103.16-124.69) | 105.82 (96.35-115.29) | 0.194 | **120.94 (109.96-131.92)** ‡ | **124.97 (115.41-134.53)** † | 0.529 | **123.31 (112.19-134.43)** † | **128.37 (118.66-138.08)** † | 0.432 |
| **SPEED (m/s) - post** | **127.46 (115.56-140.59)** | **113.26 (104.12-123.20)** | **0.037** | 128.23 (115.98-141.77) | **128.97 (118.72-140.10)** † | 0.919 | 128.31 (116.18-141.71) | **127.07 (116.78-138.28)** † | 0.864 |
| **DISTANCE (meters)** | 428.28 (383.1-473.47) | 408.97 (367.73-450.20) | 0.446 | **426.17 (379.82-472.51)** | **516.03 (474.64-557.42)** † | <0.001 | 441.47 (394.51-488.44) | **484.26 (441.37-527.16)** † | 0.119 |
| **DINAMO (kilograms, kg) - RIGHT** | 26.47 (23.24-29.70) | 26.40 (22.38-30.43) | 0.981 | **22.02 (18.25-24.94)** | **28.35 (23.57-31.75)** | **0.002** | **27.04 (22.88-29.74)** † | 29.54 (24.46-32.69) | 0.218 |
| **DINAMO (kg) - LEFT** | 25.95 (21.99-29.91) | 24.20 (20.31-28.10) | 0.339 | **22.02 (17.97-26.08)** ‡ | 25.70 (21.76-29.64) | 0.231 | 27.07 (22.96-31.18) | 25.08 (21.09-29.08) | 0.316 |
| **BERG scale** | 54.78 (51.15-58.41) | 51.74 (48.59-54.89) | 0.139 | 54.99 (51.31-58.68) | **54.74 (51.56-57.92)** ‡ | 0.903 | 54.19 (50.47-57.92) | **54.39 (51.18-57.61)** ‡ | 0.923 |

*Values represent adjusted estimated means (95% CI). P values correspond to between-group comparisons at each visit derived from the adjusted models. Symbols (†, ‡) indicate within-group changes relative to baseline.*

*† p<0.001 (relative to visit 1). ‡ p<0.05 (relative to visit 1)*

Cognitive Assessment – Models adjusted for age, hypertension, and smoking status

|  | **PLACEBO** | **SALICORNIA EXTRACT** | ***P*** | **PLACEBO**  **N=23** | **SALICORNIA EXTRACT**  N=26 | ***P*** | **PLACEBO**  N=22 | **SALICORNIA**  **EXTRACT**  N=19 | ***P*** |
| --- | --- | --- | --- | --- | --- | --- | --- | --- | --- |
|  | **VISIT 1** | | | **VISIT 2** | | | **VISIT 3** | | |
| **Montreal Cognitive Assessment (MOCA) - Direct Score** | 21.73 (19.67-23.79) | 22.89 (21.38-24.41) | 0.271 | **21.72 (19.57-23.88)** | **24.59 (23.07-26.12)** ‡ | **0.011** | **22.88 (20.70-25.07)** | **25.65 (24.01-27.29)** † | **0.018** |
| **MOCA - Adjusted**  **(age and education)** | 7.69 (6.18-9.61) | 7.96 (6.68-9.48) | 0.779 | 7.74 (6.16-9.74) | **9.54 (8.00-11.37)** ‡ | 0.088 | 8.45 (6.71-10.66) | **10.61 (8.77-12.84)** † | 0.074 |
| **Symbol Digit Modality Test (SDMT)** | 10.01 (8.74-11.29) | 9.31(8.28-10.36) | 0.321 | 9.98 (8.61-11.34) | 10.36 (9.31-11.41) | 0.604 | 10.82 (9.41-12.23) | 10.29 (9.12-11.45) | 0.501 |
| **Five digit Test (5DIG)-flexibility** | **2.30 (0.05-7.87)** | **10.71 (4.75-19.05)** | **0.017** | **6.48 (1.23-15.88)** | **31.61 (20.64-44.90)** † | **<0.001** | **24.24 (11.98-44.77)** † | **30.97 (18.91-45.98)** † | 0.819 |
| **Five digit Test (5DIG) -inhibition** | 31.43 (15.91-46.96) | 25.68 (12.88-38.46) | 0.504 | 41.96 (24.49-59.41) | 27.20 (13.98-40.41) | 0.125 | 39.50 (21.59-57.41) | 30.24 (15.59-44.89) | 0.367 |
| **Trail Making Test part A (TMT-A)** | 11.02 (9.30-12.75) | 10.63 (9.25-12.02) | 0.672 | 11.76 (9.94-13.58) | 11.56 (10.12-12.99) | 0.836 | 11.71 (9.82-13.60) | 11.10 (9.59-12.61) | 0.558 |
| **Trail Making Test, part B (TMT-B)** | 9.92 (8.40-11.44) | 10.05 (8.76-11.34) | 0.873 | 9.84 (8.23-11.45) | 10.56 (9.27-11.85) | 0.405 | 10.42 (8.66-12.18) | 8.91 (7.50-10.33) | 0.127 |

*Values represent adjusted estimated means (95% CI). P values correspond to between-group comparisons at each visit derived from the adjusted models. Symbols (†, ‡) indicate within-group changes relative to baseline.*

*† p<0.001 (relative to visit 1). ‡ p<0.05 (relative to visit 1)*

Gait Analysis – Models adjusted for age, hypertension, smoking status, and baseline Values

|  | **PLACEBO**  **N=34** | **SALICORNIA EXTRACT**  **N=23** | ***P*** | **PLACEBO**  **N=29** | **SALICORNIA EXTRACT**  **N=22** | ***P*** |
| --- | --- | --- | --- | --- | --- | --- |
|  | **VISIT 2** | | | **VISIT 3** | | |
| **SPEED (meters per second m/s) - pre** | **109.21 (101.82-116.60)** | **121.32 (115.07-127.57)** | **0.004** | **111.83 (104.15-119.50) ‡** | **123.74 (117.29-130.19) †** | **0.006** |
| **SPEED (m/s) - post** | 115.16 (104.47-125.84) | 127.19 (118.22-136.16) | 0.053 | 118.83 (108.22-129.65) | 127.85 (118.47-137.23) | 0.153 |
| **DISTANCE (meters)** | **391.91 (356.29-427.49)** | **505.94 (475.10-536.77)** | **<0.001** | **408.45 (372.11-444.80)** | **464.28 (432.28-496.29)** ‡ | **0.008** |
| **DINAMO (kilograms, kg) - RIGHT** | **22.02 (18.92-25.12)** | **28.35 (25.23-31.47)** | **0.002** | **27.05 (23.86-30.24)** † | 29.54 (26.40-32.68) | 0.218 |
| **DINAMO (kg) - LEFT** | 24.25 (21.29-27.22) | 26.50 (23.73-29.26) | 0.229 | **28.88 (25.91-31.86)** † | 26.25 (23.41-29.07) | 0.160 |
| **BERG scale** | 52.94 (50.59-55.29) | 54.72 (52.70-56.74) | 0.187 | 52.18 (49.77-54.58) | 54.31 (52.23-56.39) | 0.663 |

*Values represent adjusted estimated means (95% CI). P values correspond to between-group comparisons at each visit derived from the adjusted models. Symbols (†, ‡) indicate within-group changes relative to the previous visit.*

*† p<0.001 (relative to visit 2). ‡ p<0.05 (relative to visit 2)*

**Cognitive Assessment – Models adjusted for age, hypertension, smoking status, and baseline Values**

|  | **PLACEBO**  **N=23** | **SALICORNIA EXTRACT**  N=26 | ***P*** | **PLACEBO**  N=22 | **SALICORNIA**  **EXTRACT**  N=19 | ***P*** |
| --- | --- | --- | --- | --- | --- | --- |
|  | **VISIT 2** | | | **VISIT 3** | | |
| **Montreal Cognitive Assessment (MOCA) - Direct Score** | 22.96 (21.32-24.60) | 24.54 (23.28-25.79) | 0.082 | 23.93 (22.25-25.61) | 25.36 (23.80-26.93) | 0.142 |
| **MOCA - Adjusted**  **(age and education)** | 8.91 (7.02-10.79) | 10.22 (8.81-11.63) | 0.193 | **9.60 (7.68-11.51)** | **11.82 (10.10-13.54)** | **0.042** |
| **Symbol Digit Modality Test (SDMT)** | 9.93 (8.57-11.28) | 10.59 (9.57-11.59) | 0.361 | 10.77 (9.37-12.18) | 10.31 (9.17-11.45) | 0.549 |
| **Five digit Test (5DIG)-flexibility** | **10.50 (1.41-28.05)** | **34.56 (20.20-52.76)** | **0.013** | **36.52 (15.88-65.63) †** | 36.58 (19.36-59.22) | 0.997 |
| **Five digit Test (5DIG) -inhibition** | 47.41 (27.46-67.35) | 37.29 (22.18-52.40) | 0.314 | 48.41 (27.93-68.89) | 41.68 (24.89-58.47) | 0.534 |
| **Trail Making Test part A (TMT-A)** | 11.33 (9.67-12.99) | 11.35 (9.98-12.73) | 0.983 | 11.21 (9.43-12.99) | 10.58 (9.09-12.08) | 0.529 |
| **Trail Making Test, part B (TMT-B)** | 10.07 (8.05-12.10) | 10.71 (9.19-12.23) | 0.530 | 10.85 (8.69-13.01) | **9.04 (7.35-10.74)** ‡ | 0.117 |

*Values represent adjusted estimated means (95% CI). P values correspond to between-group comparisons at each visit derived from the adjusted models. Symbols (†, ‡) indicate within-group changes relative to the previous visit.*

*† p<0.001 (relative to visit 2). ‡ p<0.05 (relative to visit 2)*
